# First description of the complete human xylosyltransferase-I promoter region

**DOI:** 10.1186/s12863-014-0129-0

**Published:** 2014-12-05

**Authors:** Isabel Faust, Kai Oliver Böker, Christoph Lichtenberg, Joachim Kuhn, Cornelius Knabbe, Doris Hendig

**Affiliations:** Institut für Laboratoriums- und Transfusionsmedizin, Herz- und Diabeteszentrum Nordrhein-Westfalen, Universitätsklinik der Ruhr-Universität Bochum, Bad Oeynhausen, Germany

**Keywords:** Xylosyltransferase, Promoter, Single-nucleotide variant, Microsatellite, Gene regulation

## Abstract

**Background:**

Human xylosyltransferase-I (XT-I) catalyzes the rate-limiting step in proteoglycan glycosylation. An increase in *XYLT1* mRNA expression and serum XT activity is associated with diseases characterized by abnormal extracellular matrix accumulation like, for instance, fibrosis. Nevertheless, physiological and pathological mechanisms of transcriptional XT regulation remain elusive.

**Results:**

To elucidate whether promoter variations might affect the naturally occurring variability in serum XT activity, a complete sequence analysis of the *XYLT1* promoter was performed in genomic DNA of healthy blood donors. Based on promoter amplification by a specialized PCR technique, sequence analysis revealed a fragment of 238 bp, termed *XYLT1*_238*_, which has never been described in the human *XYLT1* reference sequence so far. *In silico* characterization of this unconsidered fragment depicted an evolutionary conservation between sequences of *Homo sapiens* and *Pan troglodytes* (chimpanzee) or *Mus musculus* (mouse), respectively. Promoter activity studies indicated that *XYLT1*_238*_ harbors various transcription factor binding sites affecting basal *XYLT1* expression and inducibility by transforming growth factor-β1, the key fibrotic mediator.

A microsatellite and two single nucleotide variants (SNV), c.-403C>T and c.-1088C>A, were identified and genotyped in 100 healthy blood donors. Construct associated changes in *XYLT1* promoter activity were detected for several sequence variants, whereas serum XT activity was only marginally affected.

**Conclusions:**

Our findings describe for the first time the entire *XYLT1* promoter sequence and provide new insights into transcriptional regulation of XT-I. Future studies should analyze the impact of regulatory *XYLT1* promoter variations on XT-associated diseases.

## Background

The isoenzymes human xylosyltransferase-I and -II (XT-I/-II; EC 2.4.2.26) catalyze the key step of glycosaminoglycan biosynthesis by transferring activated UDP-xylose to selected serine residues on a proteoglycan core-protein in the golgi [1,2]. Proteoglycans are components of the extracellular matrix (ECM) responsible for tissue stabilization, hydration and efficient functionality of various signal transduction pathways [3,4].

In fibrosis, a dysbalance of matrix synthesis and degradation results in abnormal matrix accumulation, which initiates the functional loss of the affected organ. Although different origins of fibrotic tissue remodeling processes, as well as their molecular mechanisms are described, no anti-fibrotic treatment could be developed until now [5,6]. Among others, an increased *XYLT1* mRNA expression, as well as XT secretion and serum activity could be shown to be associated with a rise in myofibroblast differentiation and matrix synthesis in liver fibrosis, skin fibrosis or dilated cardiomyopathy [7-10]. Recently, dysregulations of XT have also been connected with the manifestation of osteoarthritis or disorders of ossification [11-13].

XT-I and -II, encoded by the genes *XYLT1* and *XYLT2*, are characterized by slight differences in their substrate specificities. Nevertheless, they are distinguishable by their local tissue expression patterns [14,15]. Based on the hypothesis that XT are pivotal regulators of pathobiochemistry, current studies address the analysis of their regulatory mechanisms. With regard to transcriptional control, two central key mediators, TGF-β1 and IL-1β, were identified [9,11]. Unfortunately, due to their wide-spread scope, none of these proteins represents an adequate point of therapeutic application. Thus, it is of great importance to identify additional basic pathways of XT regulation.

So far, *XYLT1* and *XYLT2* promoter regions have been initially identified and characterized. It could be demonstrated that both regions miss common eukaryotic promoter elements like a TATA- or CAAT-box, contain highly GC-rich passages upstream of the translational start site and are controlled by transcription factors of the AP- and SP-family [16,17]. Due to the highly GC-rich template, *XYLT1* promoter characterization was based on gene synthesis referring to the *XYLT1* reference sequence [GenBank Accession Number NG_015843.1]. Therefore, neither the *XYLT1* promoter conservation nor the occurrence of its sequence variants was formerly defined.

The aim of this study was to analyze whether naturally occurring promoter sequence variants like single nucleotide variants (SNV) could play an emerging role in *XYLT1* transcriptional regulation. A microsatellite and two SNV were identified and genotyped in genomic DNA of 100 healthy blood donors, although these variants exerted only marginal effects on serum XT activity. Nevertheless, the SNV c.-1088C>A significantly reduced promoter activity. In addition to SNV screening, we identified and characterized an evolutionary conserved fragment of 238 bp in the *XYLT1* promoter region which has never been described in the published *XYLT1* human reference sequence so far. In summary, this is the first study defining and characterizing the variability of the complete *XYLT1* promoter sequence in the general population which in turn extensively enlarges our insights in promoter organization and transcriptional regulation of human XT-I.

## Methods

### Study subjects and collection of blood samples

For this study, EDTA plasma and serum samples of 100 healthy blood donors (50% males; 18-60 years of age; mean age +/- SD: 36.2 +/- 13.5 years) were collected. The experimental design and research has been performed in accordance with the Declaration of Helsinki and was approved by the local ethics committee (medical faculty, Ruhr-Universität Bochum, Bad Oeynhausen, Germany). All blood donors gave their informed consent.

### DNA extraction and *XYLT1* promoter amplification

After plasma centrifugation, genomic DNA was extracted from 200 μL EDTA blood leukocytes using the Nucleo Spin Blood Kit (Macherey-Nagel). Referring to the current *XYLT1* reference sequence [GenBank Accession Number NG_015843.1], the promoter region was divided into four overlapping fragments termed D (c.-1689 to c.-1273), C (c.-1385 to c.-813), B (c.-891 to c.-215) and A (c.-372 to c. + 122). The amplification of fragments D, C and B was performed by an initial denaturation step at 95°C (15 min) followed by 35 cycles (denaturation at 95°C for 1 min, annealing at optimal annealing temperature (T_A_) for 1 min and elongation at 72°C for 1 min) and final elongation at 72°C for 15 min. All PCR and sequencing reactions were run on thermocycler T professional (Biometra). Primer sequences and T_A_ are listed in Table 1. The reaction mixture was composed of 11.15 μL water, 5.0 μL Q-Solution (Qiagen), 0.25 μL dNTPs (25 mM), 2.5 μL reaction buffer (Qiagen), 0.5 μL of each primer (25 μM), 5.0 μL template (dilution 1:5) and 0.1 μL HotStar *Taq* DNA-polymerase (Qiagen). The amplification of the GC-rich A fragment required highly specialized conditions. The used slowdown-PCR cycling conditions are described elsewhere, while the composition of the reaction mixture (15.8 μL water, 10.0 μL Q-Solution, 5.0 μL reaction buffer, 2.0 μL of each primer (2.5 μM), 1.0 μL of dATP, dTTP and dCTP (10 mM), 2.5 μL of dGTP (1 mM), 7.5 μL dc^7^GTP (1 mM; Roche), 0.2 μL HotStar *Taq* DNA-polymerase and 2.0 μL template) was adjusted [18,19].

**Table 1:** **Primers used for amplification**, **sequencing and site**-**directed mutagenesis** (**SDM**) **of** ***XYLT1*** **promoter fragments**

**Application**		**Primer sequence** **(** **5**′ → **3** **′)**	**T**_**A**_ **[°C]**
PCR and sequencing	Fragment A	CCCTGTTTCGCGGCCCCTG	*Slowdown*-PCR
		CCGGAGTCGAGGCTGCTGAA	
	Fragment B1	ATGGATGGGGAAAAGGACAC	58.8
		TGGGGGAGGAGCCGAGGGAG	
	Fragment B2	CAGTCAGGATGGGAAAGAAC	58.8
		GGTGCCAACGATGTACTAAG	
	Fragment C	AACAATCTCTTCCCACTCCC	61.2
		GGAGTTACTCAACCTTCGCA	
	Fragment D	CATGCCCGGCTAATTTTTGT	61.2
		CTTCGCATCTTGTCTGCTGT	
	Fragment *XYLT1*_238*_	ACAGGGGTGTGGGGAGGGGGCGCCGCGCGGGCCAGGCGCC	58.8
		GCTCGGGCCGCCGCCGCCGCCGCCGCCTCGGCTCGCCGCT	
Plasmid sequencing		CTAGCAAAATAGGCTGTCCC	
		CTTAATGTTTTTGGCATCTTCCA	
SDM	c.-403C>T	TCCCCCCGGCGCCTTCCCCAT**T**ACCCTCCCCTCCAGCGGGGA	
		TCCCCGCTGGAGGGGAGGGT**A**ATGGGGAAGGCGCCGGGGGGA	
	c.-1088C>A	GCTGGGAGGCTGCGGGGCCAG**A**CTTTGGGGCTTGCATCCTGC	
		GCAGGATGCAAGCCCCAAAG**T**CTGGCCCCGCAGCCTCCCAGC	

Mutated bases are marked in bold.

### Promoter sequencing, mutational analysis and genotyping

Sanger sequencing was initiated by PCR product clean-up. 5.0 μL of the PCR product were incubated with 2.0 μL of exonuclease I (NEB), as well as 2.0 μL shrimp alkaline phosphatase (Affymetrix) at 37°C for 30 min and 80°C for 15 min. 2.0 μL of the reaction mixture were added to a sequencing master mix containing 5.5 μL water, 5.0 μL Q-solution (Qiagen), 4.0 μL BigDye v3.1 sequencing premix (Life technologies), 2.0 μL 5 × BigDye sequencing buffer (Life technologies) and 1.5 μL primer (25 μM; listed in Table 1). Sequencing reaction started with a denaturation step at 95°C for 2 min and run for 30 cycles (denaturation at 95°C for 10 s, annealing at T_A_ for 10 s, elongation at 60°C for 4 min). After purification by a sephadex-G50 spin-column based protocol, sequences were analyzed by capillary electrophoresis using the Genetic Analyzer 3500 (Life technologies).

### Promoter construct cloning and insertion of SNV by site-directed mutagenesis

Construction of pGL4.10 luciferase reporter vectors containing the *XYLT1* promoter fragments c.-1639 to c.+1_Δ238*_ or c.-1031 to c.+1_Δ238*_, according to the current uncomplete reference sequence, was described previously [16]. To adjust the promoter length, we inserted the missing region *XYLT1*_238*_ using the QuikChange site-directed mutagenesis kit (Agilent). *XYLT1*_238*_ was amplified from the PCR product of the A fragment (primers are listed in Table 1) and purified by the MSB Spin PCRapace kit (Stratec). Site-directed mutagenesis cycling conditions and composition of the reaction mixture are published elsewhere [20]. To insert single nucleotide sequence variants in the synthesized vector construct c.-1031 to c.+1_complete_, site-directed mutagenesis was applied according to the manufacturer’s instructions (primers are listed in Table 1). Successful insertion of *XYLT1* promoter fragment or single base exchange was checked by amplification and sequencing of the appropriate plasmid region or direct plasmid sequencing using vector specific primers (Table 1).

### Cell culture and transient transfection of plasmids

SW1353 chondrosarcoma cells were routinely grown in RPMI 1640 medium (Life technologies), supplemented with 10% FCS (Pan biotech) and 1% antibiotic/antimycotic solution (PAA). For transfection, 180000 cells were seeded in triplicate in 6 well culture dishes and incubated for 24 h. The next day, 188 μL medium (without any supplements) were mixed with 12 μL FuGene 6 transfection reagent (Promega) and incubated at RT for 5 min. After addition of 1 μg of the appropriate pGL4.10 plasmid and 10 ng of pGL4.74, the reaction mixture was incubated for 30 min and applied to the cells. After 24 h, the cell culture medium was replaced. Where appropriate, TGF-β1 (7.5 ng/mL, Miltenyi Biotech) was added. Cells were harvested in 500 μL lysis buffer and promoter activity was analyzed after 48 h.

### Dual luciferase reporter assay

Cellular luciferase activity was assayed with the Dual Luciferase Reporter assay system (Promega) on a Lumat LB9705 luminometer (EG&G). 20 μL lysate were incubated with 100 μL LARII substrate to stimulate substrate turnover of *firefly* luciferase (encoded by the pGL4.10 vector construct upstream of the *XYLT1* promoter sequence). Addition of 100 μL Stop&Glo solution inhibited *firefly* luciferase and simultaneously induced substrate turnover of *renilla* luciferase (encoded by the co-transfected pGL4.74 vector). To calculate relative luciferase activity representing promoter activity, *firefly* luciferase activity was measured in each sample and normalized to *renilla* luciferase activity. Normalization reduced the impact of differences in efficiency of transfection or lysis.

### XT activity assay

Determination of serum XT activity was performed as described before. The method relies on incorporation of ^14^[C] D-xylose (Du Pont) into silk fibroin receptor protein. Measured disintegrations per minute (dpm) are proportional to enzymatic activity [21,22].

### *In silico* analysis

*In silico* analysis of transcription factor binding sites was carried out using the Genomatix online software suite, while sequence alignments were performed with Clone Manager 9.0 (Scientific & Educat. Software) and ClustalW (DNA Star Inc.). Evaluation of LD maps and haplotype frequencies was carried out using Haploview 4.0 (Broad Institute) [23]. Blocks were defined according to the “solid spine of LD” setting in the software.

### Statistics

Experimental data were analyzed by Mann-Whitney-U-Test using GraphPad Prism 5.0 (GraphPad Software). p values less than 0.05 were considered statistically significant. To examine whether genotype distributions fit into the Hardy-Weinberg equilibrium, a χ^2^-test was performed.

## Results

### Sequence analysis of the human *XYLT1* promoter region

To screen for natural occurring variants in the *XYLT1* promoter, we performed a preliminary sequence analysis of the genomic DNA of ten healthy blood donors. Referring to the published *XYLT1* reference sequence [GenBank Accession Number NG_015843.1], the amplification was performed by splitting the region into four fragments, A to D. Due to a high GC-content of 75%, the A fragment (c.-372 to c.+122), could exclusively be amplified by slowdown-PCR conditions. Sequencing results demonstrated that the *XYLT1* promoter region does not comprise just 1639 bp, as published earlier [16] but also harbors an additional fragment of 238 bp in between the known sequence of the A fragment. The nucleotide sequence for the A fragment has been deposited in the GenBank database [GenBank Accession Number KM079589].

**Figure 1:**
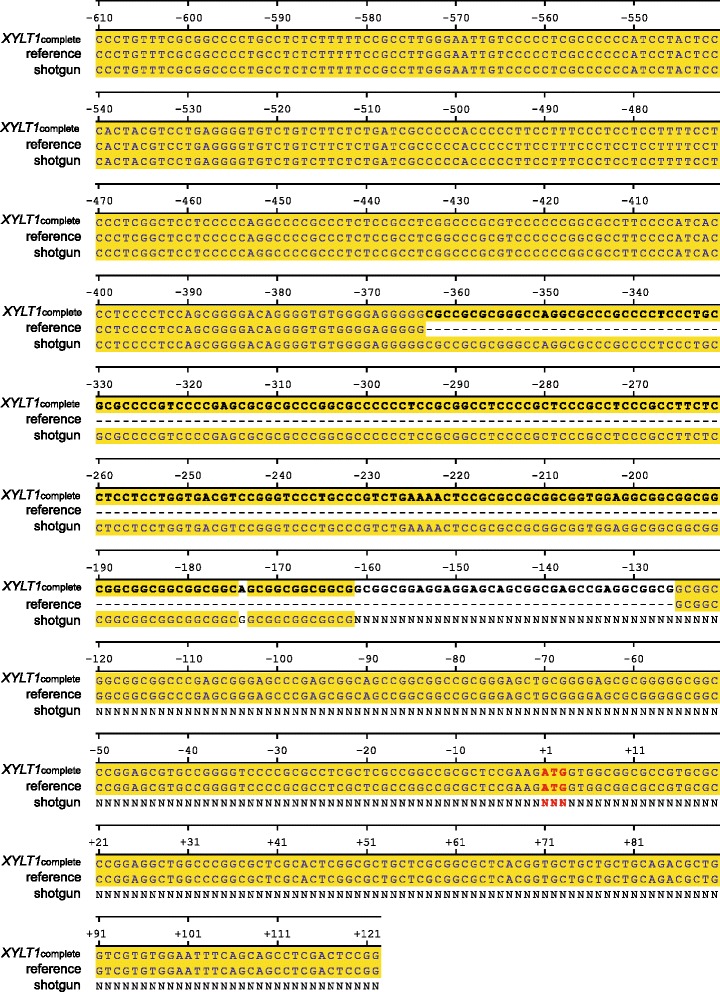
**Sequence alignment of human genomic** ***XYLT1*** **promoter sequences** **(** **c** **.-** **610 to c** **.+** **122** **).** The sequence examined in this study (*XYLT1*_complete_, [GenBank Accession Number KM079589]) was compared with the appropriate *XYLT1* promoter region of the current human reference sequence [GenBank Accession Number NG_015843.1] and a human shotgun sequence [GenBank Accession Number NW_001838365.2]. Numbers indicate the nucleotide position up- or downstream of the translation initiation site ATG (red color). Matching nucleotides are shaded in yellow, whereas *XYLT1*_238*_ (c.-363 to c.-126) is marked in bold.

The identification of this hitherto undescribed *XYLT1* promoter fragment, termed *XYLT1*_238*_, was verified by sequencing the genomic DNA of 100 healthy blood donors. All PCR products spanned approximately 700 bp, so none of the amplificates displayed the calculated fragment length of 494 bp, referring to the current *XYLT1* reference sequence [GenBank Accession Number NG_015843.1]. A sequence alignment of the reference sequence, as well as the consensus sequence of the *XYLT1* promoter sequence *XYLT1*_complete_, identified in this study, confirmed the absence of *XYLT1*_238*_ in the published reference sequence (Figure 1). In the following, base numbering depends on promoter sequence *XYLT1*_complete_, whereby the first nucleotide of the translation initiation start site was defined as c.+1 and promoter nucleotides were numbered backwards. *XYLT1*_238*_ was located at position c.-363 to c.-126. By screening data bases for complementary human DNA reference sequences, *in silico* analysis revealed the deposition of only one shotgun sequence [GenBank Accession Number NW_001838365.2] conforming to *XYLT1*_238*_. However, due to a sequence cut off at position c.-162, *XYLT1*_238*_ is merely partially listed (Figure 1).

**Figure 2:**
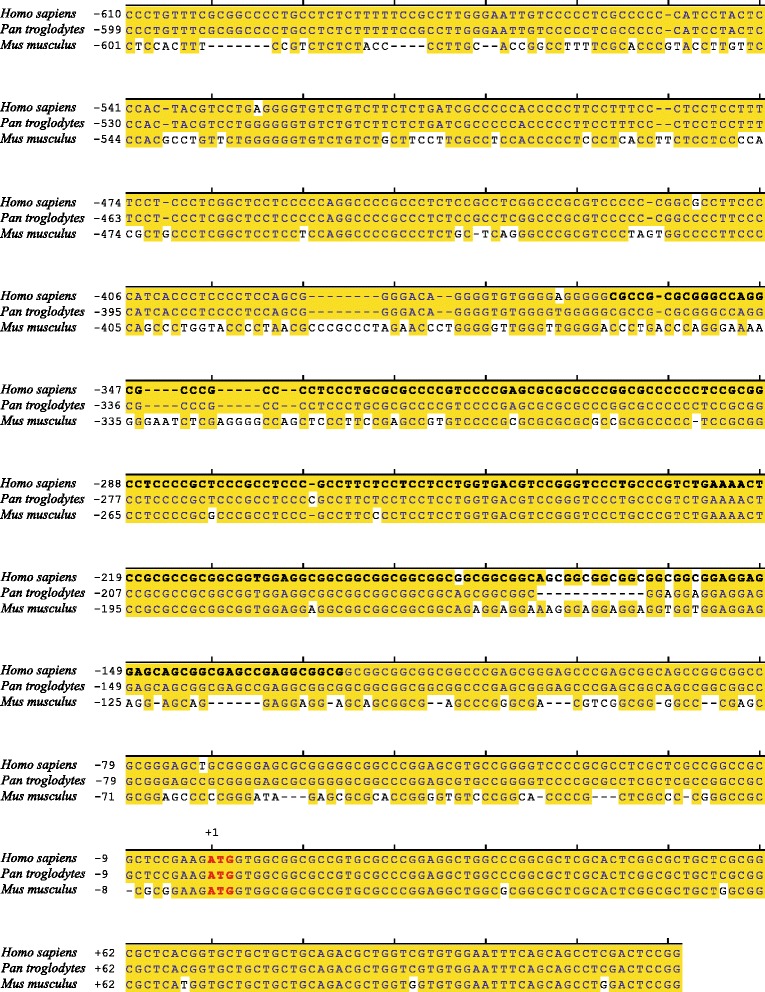
**Cross**-**species sequence alignment of the** ***XYLT1*** **promoter sequence c**.-**610 to c**.+**122.** Genomic DNA sequences of *Homo sapiens* (*XYLT1*_complete_, [GenBank Accession Number KM079589]), *Pan troglodytes* [GenBank Accession Number NC_006483.3] and *Mus musculus* [GenBank Accession Number NC_000073.6] have been compared. Numbers indicate the nucleotide position up- or downstream of the translation initiation site ATG (red color). Matching nucleotides are shaded in yellow, whereas *XYLT1*_238*_ (c.-363 to c.-126) is marked in bold.

An evolutionary species alignment of the PCR A fragment (c.-610 to c.+122) represented a strong overlap of human promoter sequence *XYLT1*_complete_ [GenBank Accession Number KM079589] with corresponding DNA fragments of related species *Pan troglodytes* [GenBank Accession Number NC_006483.3] and *Mus musculus* [GenBank Accession Number NC_000073.6]. While the compliance of the whole genomic *XYLT1* promoter sequences between *Homo sapiens* (c.-1877 to c. + 1) and *Pan troglodytes* assessed as 98%, the compliance of *XYLT1*_238*_ (c.-363 to c.-126) was 93% (Figure 2). Corresponding sequence conformity of *Homo sapiens* and *Mus musculus* sequences amounted to 58% in the whole and to 77% concerning *XYLT1*_238*_.

### Characterization of the *XYLT1* promoter fragment *XYLT1*_238*_

**Figure 3:**
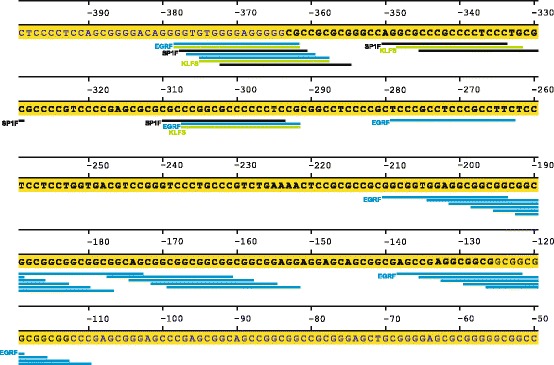
**Localization of transcription factor binding sites in** ***XYLT1***_238*_ **and appropriate flanking regions.** Numbers indicate the nucleotide position downstream of the translation initiation site (ATG). *XYLT1*_238*_ (c.-363 to c.-126) is marked in bold, while transcription factor binding sites are highlighted in black (SP1F), blue (EGRF) or green (KLFS).

*XYLT1*_238*_ displays a GC content of 84.9% and harbors a variable microsatellite region (c.-201 to c.-148). To identify putative transcription factor binding sites, an *in silico* analysis was performed. Search criteria were restricted to families of transcription factors, which have been discussed to modulate XT or which are known mediators of TGF-β1. As indicated in Figure 3, several transcription factor binding sites of transcriptions factor families SP1F (GC-box factors, specificity protein1), EGRF (early growth response/nerve growth factor induced protein C and related factors) and KLFS (krueppel like transcription factors) were identified.

**Figure 4:**
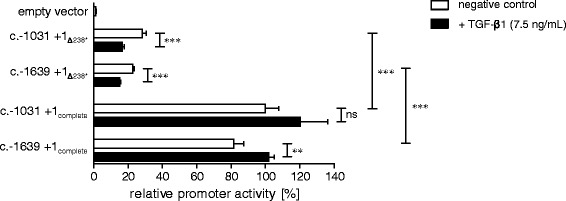
**Influence of** ***XYLT1***_238*_ **on promoter activity.** Changes in relative promoter activity of pGL4.10 *XYLT1* promoter cloning constructs were determined in response to insertion of *XYLT1*_238*_ by site-directed mutagenesis. Promoter activities are expressed relative to the activity of construct c.-1031 to c.+1_complete_, which was defined as 100%. Plasmids were transfected into SW1353 cells and promoter activity was determined in lysates of untreated (white bars) or TGF-β1 induced (black bars) cells by dual luciferase assay. Values are means ± SEM of triplicates from at least two or three independent experiments. ns = not significant; **p < 0.01; ***p < 0.001 (Mann-Whitney U-test).

To evaluate the influence of *XYLT1*_238*_ on promoter activity, appropriate promoter luciferase constructs were cloned by insertion of *XYLT1*_238*_ in existing *XYLT1* promoter coding luciferase vectors using site-directed mutagenesis. These plasmid constructs were originally synthesized for initial promoter characterization [16] and include the entire, but as we could define incomplete, promoter region c.-1639 to c.+1_Δ238*_ or the most active promoter segment c.-1031 to c.+1_Δ238*_ according to the *XYLT1* reference sequence [GenBank Accession Number NG_015843.1]. After plasmid transfection into SW1353 chondrosarcoma cells and subsequent performing of a dual luciferase assay, promoter activity was shown to become significantly upregulated by insertion of *XYLT1*_238*._ Comparing promoter activity of constructs c.-1639 to c.+1_Δ238*_ and c.-1031 to c.+1_Δ238*_ to the vectors c.-1639 to c.+1_complete_ and c.-1031 to c.+1_complete_ revealed a significant increase of 22.6% (±0.9% SEM) to 81.6% (±5.6% SEM) or 28.3% (±2.2% SEM) to 100.0% (±1.1 %SEM), respectively (Figure 4, white bars). To analyze the putative inducibility of the *XYLT1* promoter activity by TGF-β1, SW1353 cells were transfected as described above, followed by incubation with cell culture medium supplemented with TGF-β1 for 48 h. While no increase in promoter activity was detected for constructs c.-1639 to c.+1_Δ238*_ and c.-1031 to c.+1_Δ238*_ in comparison to untreated controls, promoter activity of constructs c.-1639 to c.+1_complete_ and c.-1031 to c.+1_complete_ enlarged from 100% (±1.1% SEM) to 120.2% (±16.2% SEM) or 81.6% (± 5.6% SEM) to 101.7% (±3.2% SEM), respectively (Figure 4, black bars).

### SNV analysis

In addition to identification of *XYLT1*_238*_, genotyping of the complete promoter region revealed two SNV. Allele frequencies of c.-403C>T and c.-1088C>A were determined in DNA samples of 100 healthy blood donors (Table 2). All genotype distributions accorded with the Hardy-Weinberg equilibrium, whereas haplotype analysis depicted a weak linkage disequilibrium (D’ = 0.85; *r*^2^ = 0.49; haplotypes Table 3).

**Table 2:** **Allele frequencies of** ***XYLT1*** **promoter SNV detected in healthy blood donors**

**SNV**	**rs ID**	**Allele**	**Frequency**
c.-403C>T	rs118030014	C	104/200 (0.52)
		T	96/200 (0.48)
c.-1088C>A	rs59423557	C	124/200 (0.62)
		A	76/200 (0.38)

**Table 3:** **Estimated** ***XYLT1*** **haplotypes detected in healthy blood donors**

**Haplotype**	**c**.-**403**	**c**.-**1088**	**Frequency**
1	C	C	98/200 (0.492)
2	T	A	70/200 (0.352)
3	T	C	26/200 (0.128)
4	C	A	6/200 (0.028)

**Figure 5:**
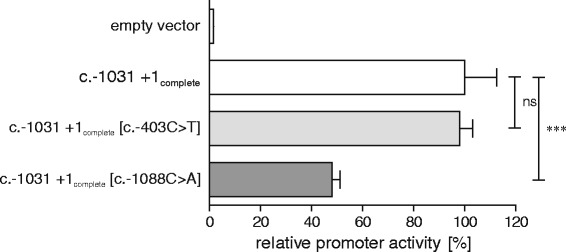
**Influence of SNV on promoter activity.** Changes in relative promoter activity of pGL4.10 *XYLT1* promoter cloning construct c.-1031 to c.+1_complete_ were determined in response to SNV-associated base exchange by site-directed mutagenesis. Promoter activities are expressed relative to the activity of construct c.-1031 to c.+1_complete_, which was defined as 100%. Plasmids were transfected into SW1353 cells and promoter activity was determined by dual luciferase assay. Values are means ± SEM of triplicates from at least two or three independent experiments. ns = not significant; ***p < 0.001 (Mann-Whitney U-test).

By performing an *in silico* analysis, it could be demonstrated that the base exchange c.-1088C>A entails the presence of a SMAD3 transcription factor binding site (reverse strand; 5′-G**T**CTGG-3’). After selective mutation of the cloning luciferase vector c.-1031 to c.+1_complete_ by site-directed mutagenesis according to the SNV genotype, SNV associated changes in promoter activity were analyzed (Figure 5). c.-403T did not exert any influence on promoter activity, whereby the mutation c.-1088A was followed by a significant reduction in activity to 48.1% (± 3.0% SEM). Contrarily, quantification of serum XT activity did not reveal any association between SNV genotype and serum XT activity (data not shown).

### Identification and characterization of a microsatellite in the *XYLT1* promoter region

**Figure 6:**
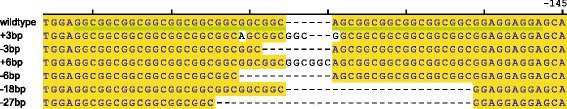
**Microsatellite sequence variants.** Naturally occurring variants of the *XYLT1* promoter region c.-211 to c.-145 are illustrated. Numbering c.-145 indicates the nucleotide position upstream of the translation initiation site, whereas numbers on the left indicate the tandem-repeat pattern. Matching nucleotides between variants are shaded in yellow. The microsatellite locus is highlighted in green.

*XYLT1*_238*_ characterization also uncovered a variable microsatellite (c.-201 to c.-148). Genotyping of 100 healthy blood donors revealed that 55% of blood donors were heterozygous carriers of this microsatellite, while 45% were homozygous carriers. In addition to the wildtype, five homozygous variants of the microsatellite were identified, distinguishable by different numbers of tandem repeats (GGX)_n_ (Figure 6). Allele frequencies are listed in Table 4. The more tandem repeats were coded in the microsatellite variant, the more EGR-1 transcription factor binding sites are strung together (Figure 3). Due to sequence chromatogram overlaps, heterozygous genotypes were definable neither by Sanger nor by pyro sequencing.

**Table 4:** **Allele frequencies of homozygous microsatellite variants detected in healthy blood donors**

**Homozygous microsatellite variant**	**Frequency**
wildtype	27/45
+ 3 bp	5/45
- 3 bp	2/45
+ 6 bp	1/45
- 6 bp	9/45
- 18 bp	1/45

**Figure 7:**
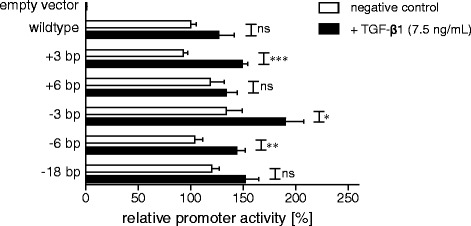
**Influence of microsatellite variants on promoter activity.** Changes in relative promoter activity of pGL4.10 *XYLT1* promoter cloning construct c.-1031 to c.+1_complete_ (wildtype) were determined in response to insertion of microsatellite variants by site-directed mutagenesis. Promoter activities are expressed relative to the activity of construct c.-1031 to c.+1_complete_, which was defined as 100%. Plasmids were transfected into SW1353 cells and promoter activity was determined in lysates of untreated (white bars) or TGF-β1 induced (black bars) cells by dual luciferase assay. Values are means ± SEM of triplicates from at least two or three independent experiments. ns = not significant; *p < 0.05; **p < 0.01; ***p < 0.001 (Mann-Whitney U-test).

Referring to SNV analysis, we also tested the influence of microsatellite variants on promoter activity. For this purpose, variants were cloned into the luciferase promoter construct c.-1031 to c.+1_complete_ by site-directed mutagenesis. Determination of relative promoter activity by dual luciferase assay in SW1353 cells depicted no dependency of tandem repeat length on promoter activity (Figure 7). Nevertheless, construct associated changes in promoter activity were detected. In addition to basal promoter activity, inducibility of microsatellite variant constructs by TGF-β1 was analyzed. Although the promoter activity of all constructs was significantly increased, no obvious trend in construct associated promoter activity difference was identifiable.

## Discussion

The aim of this study was to figure out transcriptional regulation mechanisms of XT-I. Therefore, the genomic DNA of healthy blood donors was screened for sequence variants in the human *XYLT1* promoter region to determine their influence on the naturally occurring variability of serum XT activity and to evaluate the promoter conservation. XT-I, the key enzyme of proteoglycan synthesis, is not only upregulated in the case of fibrotic remodeling, but is also associated with osteoarthritis and ossification disorders [11,12,24]. The clinical relevance of XT-I is based on its capability to represent the proteoglycan turn-over rate as a serum biomarker [25]. Hence, an increasing knowledge of its transcriptional regulation pathways will contribute to the elucidation of underlying pathomechanisms.

At present, only two publications concern the human *XYLT1* promoter region. One of them describes the initial promoter identification and characterization. Due to the high GC content, cloning was based on gene synthesis of the first 600 bp upstream of the translational start codon according to the current *XYLT1* reference sequence [GenBank Accession Number NG_015843.1] [16]. Another research project deals with the investigation of *XYLT1* promoter activity regulation by IL-1β. As indicated, promoter deletion constructs were cloned by single fragment promoter amplification [26].

In this study, the first sequence analysis of the complete *XYLT1* promoter region was performed. Surprisingly, we were not able to fully reproduce the current *XYLT1* reference sequence [GenBank Accession Number NG_015843.1] but rather found out that the *XYLT1* promoter is larger than expected and harbors a hitherto undescribed fragment of 238 bp, termed *XYLT1*_238*_ [GenBank Accession Number KM079589]. To confirm this, we performed an *in silico* analysis revealing undeniable compliance of one single human shotgun sequence [GenBank Accession Number NW_001838365.2]. Interruption of the shotgun sequence reflects severity in amplification of this promoter fragment. In this study, successful amplification was only achievable by choosing slowdown-PCR conditions [18,19]. Molecular circumstances causing difficulties in amplification are uncertain. Speculatively, DNA secondary structures are involved in complex DNA assembly. We identified a potential structure of a G-quadruplex in the *XYLT1* promoter (c.-387 to c.-364). G-quadruplex elements are known to influence transcriptional regulation and may be loosened by addition of PCR amplification additives *in vitro* [27,28]. Here, dc^7^GTP was used to minimize hogsteen base pairing in the G-quadruplex structure [29].

However, a high percentage match was detected by a cross-species alignment of the complete human *XYLT1* promoter sequence *XYLT1*_complete_ described here with sequences of *Pan troglodytes* [GenBank Accession Number NC_006483.3] and *Mus musculus* [GenBank Accession Number NC_000073.6]. Based on the evolutionary similarity of *Homo sapiens* and *Pan troglodytes* genomes [30], the reported promoter sequence *XYLT1*_complete_ becomes strongly reinforced. Thus, we suggest a revision of the current *XYLT1* reference sequence.

Characterization of *XYLT1*_238*_ demonstrated a critical influence on basal transcriptional activity, as well as inducibility of XT-I by TGF-β1. Until now, studies have described an induction of *XYLT1* mRNA expression in fibrotic tissues or after incubation of cultivated cells with TGF-β1 [9,10], whereas induction of promoter activity has remained elusive. Hence, this is the first study unravelling transcriptional mechanisms of XT-I induction. Several mediators like, for instance, transcription factors of the EGRF-, SP1- or KLFS-family were identified to regulate transcription. They all are associable with the manifestation of fibrotic disorders [31,32]. The most frequent transcription factor binding site in *XYLT1*_238*_, which was elicited *in silico*, binds EGR1 (early growth response protein 1). EGR1 functions as a downstream mediator of TGF-β1 and is responsible for a SMAD-independent increase in collagen mRNA expression in systemic sclerosis [33,34].

Sequence analysis of the genomic DNA of 100 healthy blood donors revealed the occurrence of a microsatellite as well as two SNV in the *XYLT1* promoter region, and points to a strong conservation. This hypothesis is confirmed by the description of only a few SNV, representing risk factors for proteoglycan-associated pathologies like diabetic nephropathy, and two extremely rare, naturally occurring defects in the *XYLT1* gene so far [24,35,36]. The homozygous missense mutation c.1441C>T (p.R481W) was elucidated to evoke functional enzymatic alterations causing intellectual disability and dwarfism [12]. In addition, five *XYLT1*-mutations were identified in patients suffering from Desbuquois dysplasia type 2, which is defined by pathological ossification [13]. Therefore, strong conservation of promoter and protein-coding genomic sequences of *XYLT1* emphasizes its important physiological function.

In terms of the detected, genetically unlinked, SNV c.-403C>T and c.-1088C>A, a significant impairment of promoter activity was associated with homozygous nucleotide exchange c.-1088A. As indicated by *in silico* analysis, the mutation suggests generation of a SMAD3 transcription binding site. Nevertheless, SMAD3 is a cytoplasmatic mediator of TGF-β1 and should induce rather than reduce *XYLT1* promoter activity [37]. Thus, the effect of c.-1088A on promoter activity should be further verified. Contrarily, no association between SNV genotypes and changes in serum XT activity of healthy blood donors was obtained. Hence, the identified SNV might contribute to differences in *XYLT1* mRNA level but did not impact enzyme activity. It must be further investigated if changes in promoter activity correlate with changes in mRNA levels. Besides, XT-I may only marginally contribute to cumulative serum XT activity. This hypothesis is underlined by studies of Condac *et al*., who recently postulated that XT-II is the predominant XT-isoenzyme in serum [38].

Concerning the microsatellite in the *XYLT1* promoter region, five homozygous variants were identified in addition to the wildtype genotype. Microsatellites consisting of (GGX)_n_ tandem repeats are quite common in the human genome, especially in 5′ untranslated regions [39]. Nevertheless, it has to be emphasized that 55% of screened sequences were heterozygous, whereby corresponding allele types could not be resolved by standard sequencing. Future studies should deal with the exact genotyping. We further investigated the influence of the microsatellite variants on transcriptional regulation. Referring to promoter activity studies, a construct dependent variability of promoter activity was detected. However, a dependency of tandem repeat count was detected neither to basal nor TGF-β1 induced expression. Similar results were obtained by Akai *et al*., characterizing a microsatellite in the promoter region of collagen type 2 (*COL1A2*) [40]. Whether *XYLT1* gene expression is significantly influenced by microsatellite variants *in vivo* was not part of this study, due to inappropriate cohort size, and needs to be further evaluated.

## Conclusions

In summary, we describe here the first sequence analysis of the human *XYLT1* promoter region. Based on our findings, we propose a revision of the current human *XYLT1* promoter reference sequence. Our results provide new insights into transcriptional XT-I regulation. In future, genotyping of patients suffering from XT-associated diseases might uncover a potentially link between pathologic XT regulation and *XYLT1* promoter sequence variants.

### Availability of supporting data

The data set supporting the results of this study is included within the article. Genotypic data are available upon request depending on a signed declaration of exclusive research purpose.

## Abbreviations

ECM: Extracellular matrix
EGRF: Early growth response/nerve growth factor induced protein C and related factors
KLFS: Krueppel like transcription factors
SDM: Site-directed mutagenesis
SNV: Single nucleotide variant
SP1F: *GC*-box factors, specificity protein1
TGF-β1: Transforming growth factor-β1
XT: Xylosyltransferase
